# Detection of breeding signatures in wheat using a linkage disequilibrium-corrected mapping approach

**DOI:** 10.1038/s41598-021-85226-1

**Published:** 2021-03-09

**Authors:** Said Dadshani, Boby Mathew, Agim Ballvora, Annaliese S. Mason, Jens Léon

**Affiliations:** 1 Institute of Crop Science and Resource Conservation (INRES), Plant Breeding, University of Bonn, Bonn, Germany; 2 Bayer CropScience, Monheim am Rhein, Germany

**Keywords:** Genetics, Agricultural genetics, Genetic interaction, Genetic linkage study, Genetic markers, Genome, Genomics, Genotype, Plant breeding, Plant genetics, Polyploidy, Population genetics, Quantitative trait, Natural variation in plants, Plant breeding, Plant development, Plant domestication, Agricultural genetics, Field trials, Plant genetics, Plant hybridization, Polyploidy in plants, Plant sciences, Plant molecular biology

## Abstract

Marker assisted breeding, facilitated by reference genome assemblies, can help to produce cultivars adapted to changing environmental conditions. However, anomalous linkage disequilibrium (LD), where single markers show high LD with markers on other chromosomes but low LD with adjacent markers, is a serious impediment for genetic studies. We used a LD-correction approach to overcome these drawbacks, correcting the physical position of markers derived from 15 and 135 K arrays in a diversity panel of bread wheat representing 50 years of breeding history. We detected putative mismapping of 11.7% markers and improved the physical alignment of 5.4% markers. Population analysis indicated reduced genetic diversity over time as a result of breeding efforts. By analysis of outlier loci and allele frequency change over time we traced back the 2NS/2AS translocation of *Aegilops ventricosa* to one cultivar, “Cardos” (registered in 1998) which was the first among the panel to contain this translocation. A “selective sweep” for this important translocation region on chromosome 2AS was found, putatively linked to plant response to biotic stress factors. Our approach helps in overcoming the drawbacks of incorrectly anchored markers on the wheat reference assembly and facilitates detection of selective sweeps for important agronomic traits.

## Introduction

Wheat has played an essential role in the history of human civilization for centuries, and is currently the third largest staple crop, contributing about 20% of total dietary calories and proteins worldwide^1^. It is predicted that by 2050 the production of wheat has to increase by 18% in order to prevent global food insecurity^2^. Moreover, amplified by global climate change, detrimental environmental conditions such as heavy heatwaves followed by repeated precipitation extremes (heavy rain) have had dramatic negative effects on yield during the last few years^3^, and this may also compromise future food security. Triggered by the Green Revolution (GR), global wheat productivity increased dramatically in the last century (Shiferaw 2012). Main drivers of the GR were implementation of modern and efficient productions systems and breeding of high yielding wheat varieties^4^. During the last decades, the productivity of wheat in Western Europe persistently increased, making this area one of the highest-yielding regions in the world: Europe is a major net exporter of wheat^5^. However, in recent years the productivity of wheat in high-yielding regions reached a plateau. It is assumed that a biophysical yield ceiling for wheat determines the productivity of wheat in the high-yield countries of Northwest Europe (Grassini et al. 2013). Genetic bottlenecks due to past breeding events are recognized as major impediments to crop improvement^6,7^. Along with the threat of global climate change, the adverse effects of loss of genetic diversity are a major threat to global food security^8,9^.

Population genetics and genome-wide association studies (GWAS) are comprehensive approaches to assess genomic diversity and detect signatures of past or ongoing selection in breeding at the molecular level^10–12^. Previously, multiple population genomics approaches have been applied to investigate signatures of selection and domestication in plant and livestock breeding as well as for evolutionary or natural selection studies^10,13^. In wheat, several local patterns of low genetic variation, denoted as “selective sweeps”, as a consequence of strong directional selection processes have been reported with respect to flowering time and phenology^14^. Other selective sweeps have been identified as result from introgressions of resistance genes from relatives of wheat with lower ploidy levels into hexaploid wheat followed by selection for these loci^15–20^. Generally, the detection of loci under selection during crop improvement can contribute to more targeted breeding efforts and the opportunity to improve genomic selection models^14,21^.

Association panels which consist of unrelated genotypes with high genetic diversity offer an ideal basis for GWAS by linking molecular marker information with phenotypic information to uncover genes controlling phenotypic variation^22,23^. Additionally, association panels incorporating historically important cultivars and modern cultivars are also valuable sources of information on the breeding process and breeding signatures. Historically, the application of available genomic tools in hexaploid wheat has lagged behind their use in other cereals such as maize and rice^24^. This is mainly due to the complexity of the wheat genome: wheat is an allohexaploid with three closely related subgenomes, a large genome size of (~ 16 Gb) and > 85% repetitive element content^25^. However, analyzing a set of winter wheat cultivars representing 50 years of German breeding history, Lichthardt et al. (2020) were able to identify a co-evolution effect determining sink and source allocation in plants which consequently affected grain yield potential. Large-scale field experiments with the same association panel also revealed that modern cultivars consistently outperform older varieties with respect to yield parameters, grain quality, nutrient use efficiency and disease resistance (Voss-Fels et al. 2019). Studying changes of allele frequency within these panels in the course of breeding history allows us to trace the origin of beneficial alleles and thus identify valuable sources of genetic variation that may have been neglected during the breeding process. By identification and selection of genotypes containing alleles of interest these alleles can be re-introduced into modern cultivars^26^.

GWAS is based upon the principle of linkage disequilibrium (LD), the nonrandom association of alleles at different loci. LD is highly affected by population structure, which is a potential confounding factor in all genetic association studies by the existence of differing levels of genetic relatedness in populations^27–29^. Hence, understanding population structure in an association panel is an essential requirement before undertaking GWAS^30^. Multiple approaches are applied in order to correct for population structure such as Principal Components Analysis (PCA), Kinship analysis (K) and admixture proportion inference analysis (structure or admixture analysis)^31–33^. Another prerequisite to efficiently performing GWAS is the availability of accurate (high-density) genetic or physical maps of the applied markers: preferably a reference genome sequence. Along with the presentation of the reference wheat genome assembly IWGSC published a linear reference genome sequence of hexaploid wheat with RefSeq v1.0^25^. However, as the number of available genome sequences steadily increases in many crops, it is becoming more and more evident that the genome of a single genotype is not sufficient to cover the large amount of presence/absence variations or structural variants (SVs) existing within a species^34,35^. The construction of pan-genomes covering a “core” genome and “dispensable” genome containing genes that are not present in one or more genomes of the same species is hence desirable to increase the accuracy of downstream genomic analysis^36^.

Association panels incorporating historically important cultivars and modern cultivars are an ideal platform to study the process of breeding from historical perspective by detection of loci that were subjected to selection in the frame of breeding process. On the other side combining GWAS and outlier loci analysis allows detection of beneficial alleles that were neglected during the selection process of modern cultivars. The aim of the present study is to estimate the potential of a LD-corrected wheat map for use in GWAS approaches and for detection of signatures that are related to the breeding progress of German cultivars. The detection of loci under selection during breeding process can contribute to more targeted breeding efforts and the opportunity to improve breeding by application of genomic selection models.

## Materials and methods

### Plant material

The diversity panel utilized in this study comprised 221 bread wheat cultivars, including 165 German cultivars and 56 bread wheat accessions representing global genetic diversity with cultivars from Europe, USA, Mexico, India and Australia (Supplementary Table S1). The German cultivars represented 50 years of German breeding history of bread wheat from 1966 to 2016. These cultivars were selected based on their economic and historic importance. The composition of the utilized population was described in Voss-Fels, et al.^37^ and Lichthardt, et al.^38^. Seeds of the bread wheat cultivars were provided by Leibniz Institute of Plant Genetics and Crop Plant Research (IPK) by accepting the terms and conditions of the Standard Material Transfer Agreement (SMTA) of the International Treaty on Plant Genetic Resources for Food and Agriculture (http://www.fao.org/plant-treaty).

### Genotyping and data processing

The accessions of the diversity panel were genotyped at SGS TraitGenetics GmbH (Gatersleben, Germany) using the Infinium iSelect 15 K single nucleotide polymorphism (SNP) bead array, which comprises 13 006 polymorphic loci^39^, as well as by the 135 K Axiom Exome Capture Array carrying 136 780 SNP markers^38^ .

The alignment tool Bowtie2 version 2.3.4.3^40^ was used to align the sequence of the markers to the reference wheat genome assembly RefSeq v1.0^25^. Following alignment, filtering was applied in order to detect the best assignment/anchorage to a physical position on the reference genome using the default criteria of Bowtie 2.3.4.3^40^: (1) unique mapping to an unambiguous locus; (2) maximum 1 bp mismatch to the marker sequence, and (3) markers with multiple alignment options were discarded if the second-best hit showed < 3 bp mismatch to the marker sequence (i.e. markers with 2 or more hits (loci) were discarded if there was not at least 3 bp difference between the best and second-best hit). Monomorphic markers were discarded as well as SNPs with MAF (Minor Allele Frequency) less than 5% and more than 5% missing data.

**Figure 1:**
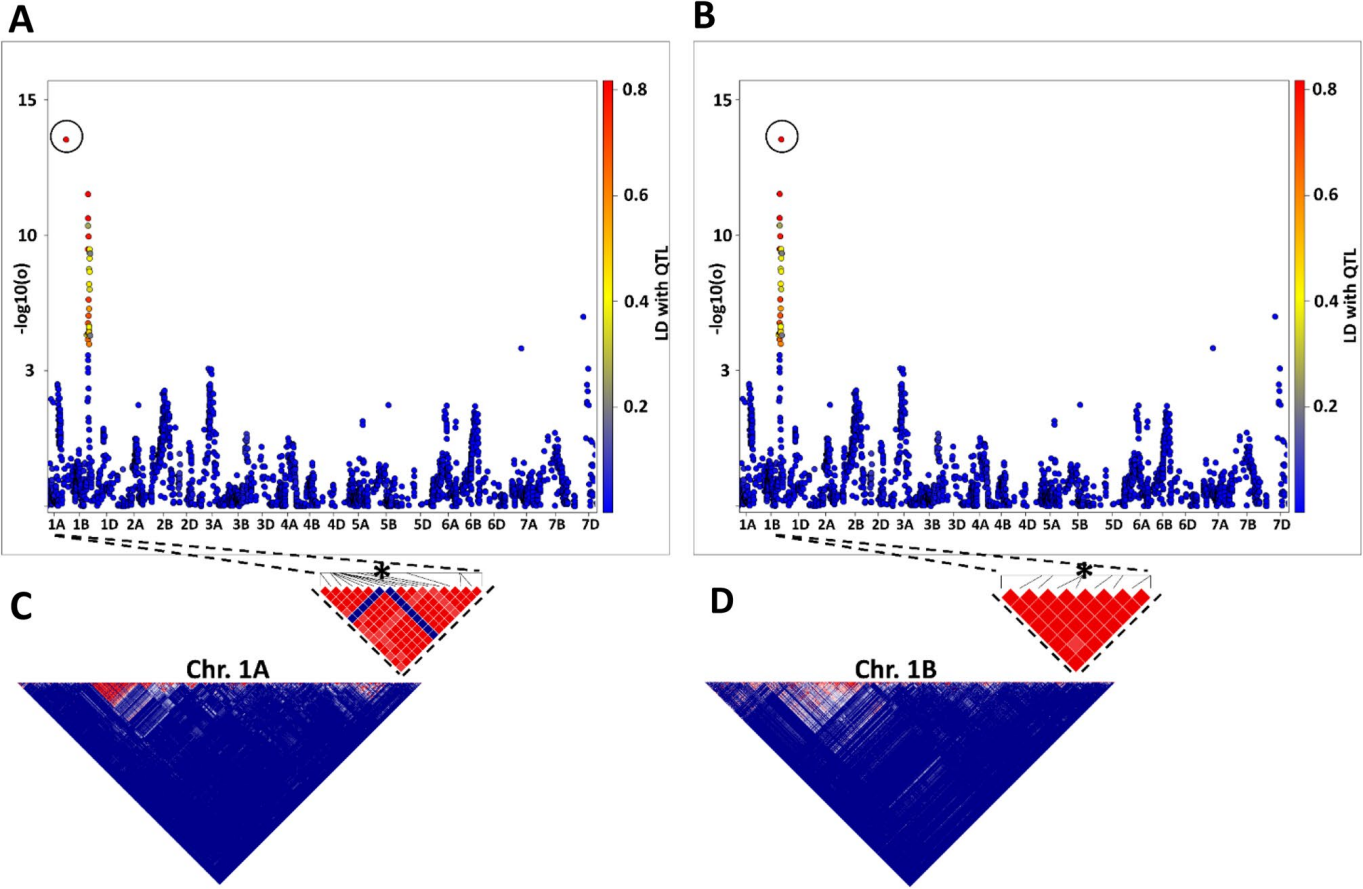
Manhattan plot of genome-wide association study (GWAS) on the dummy trait and LDHeatmap chart showing misplaced locus (**A**) and LD-based corrected position of the QTL (**B**). *P* values are shown as  − log10, circles indicate single SNP markers, colors of the circles stand for the R^2^ LD-value of the main QTL with other markers. (**C**) The LDHeatmap with focus on the misplaced/false locus of the main QTL on chromosome 1A and (**D**) the LD-corrected locus on chromosome 1B. The position of the SNP is highlighted with an asterisk.

Further filtering was applied for the markers obtaining from the Axiom array following manufacturer’s recommendation^41^ and other reports^42,43^. Consequently, only markers from the category “PolyHighResolution” and “Off-Target Variants” (OTV) showing distinct clustering of genotype calls were considered for the successive steps of filtering process. Additionally, two cluster quality control metrics, FLD (Fisher’s Linear Discriminant) and HomFLD (Homozygote Fisher's Linear Discriminant), which is a version of FLD computed for the homozygous genotype clusters, were utilized as stringent criteria. Accordingly, the minimum requirements for the corresponding individual FLD and HomFLD, were set to ≥ 4 and ≥ 8, respectively. Some markers showed high LD with markers on other chromosomes or with distant regions on the same chromosome, but no or low LD with nearby markers on the same chromosome (Fig. 1). We improved the map by following the three steps described by Utsunomiya, et al.^44^. First, genome-wide LD between each pair of SNPs was calculated using Plink v1.9^45^. Second, a table of ambiguous SNPs with low LD (r^2^ ≤ 0.5) with other markers of the same chromosome and high LD (r^2^ > 0.5) with SNP on other chromosomes was created. Third, for each marker in this list the second-best alternative alignment, confirming the region indicated by high LD, was selected as the alternative physical position. Ultimately, 24,216 informative SNP marker with defined physical positions remained for further analysis. Missing markers were imputed using the java tool LD-kNNI^46^, which is based on k-nearest neighbors imputation (kNNI) taking the LD between SNPs into account, when choosing the nearest neighbors.

### Statistical analysis

The LD between markers across chromosomes was estimated using Tassel program v.5.2.61^47^. The “prcomp” function of R was applied for calculation of marker-based principal components. The “K-means” function in the “*stats*” package in R v. 4.0.0 RC Team^48^ was used to classify the genotypes of the association panel into clusters by application of the Bayesian information criterion (BIC) as the statistical measure of goodness of fit. The R package “*adegenet”* v 2.1.2^49^ was used for the Discriminant Analysis of Principal Components (DAPC), which is a frequently used approach to identify and to describe clusters of individuals. DAPC facilitates the detection of genetic variation between groups and within-groups and yields synthetic variables which maximize between-group diversity while minimizing within-group diversity.

Additionally, the genetic structure of the diversity panel and the German cultivars was estimated separately, by using a Bayesian model-based clustering method implemented in STRUCTURE software v.2.3.4^50,51^. For this, as required by the software, marker pruning was applied by removing markers with high LD (R^2^ > 0.7) by using Plink v1.9^45^. A set of 5 681 markers remained after the pruning process. The length of the burn-in period and the number of Markov chain Monte Carlo (MCMC) iterations after burn-in were set 50,000 and 100,000, respectively. To choose the best estimate of number of clusters (K), the hypothetical number of K was set from 1 to 10. The number of replications for the MCMC run was ten times for each K.

Based on the best number of K estimated by the software STRUCTURE, Nei’s estimator of pairwise F_ST_^52^ for all pairs of sub-populations was calculated using the function “pairwise.fst” included in the “*adegenet*” package^49^. Outlier locus detection was performed by using the Windows version of ‘*BayeScan’* v2.1^53^. The Bayesian method of *BayeScan* estimates F_ST_ for each SNP locus to perform a genomic scan for outlier F_ST_ values. The following conditions were set for the BayeScan analysis to detect highly significant SNPs affected by the breeding history: the number of MCMC iterations were set to 50,000, pilot run length to 10,000 and additional burn-in to 30,000. SNP-based haplotype analysis was performed using Haploview 4.2^54^. Heat maps of pairwise LD between markers were plotted using the R package “*LDheatmap*” version 0.99-7^55^. The SNP-density plot was produced using the R package “*CMplot”*. The blast server of EnsemblPlants http://plants.ensembl.org/Triticum_aestivum/Tools/Blast (Howe et al. 2020) was used to detect genes which were identified as outlier loci in the context of breeding history.

### GWAS analysis

A genome-wide association study (GWAS) was performed using the multi-locus mixed linear model (MMLM-P + K) in SAS 9.4 (2015) taking into account population structure (P-matrix) and identity-by-state (IBS) kinship coefficient (K-matrix) between each individual. Iteratively, the forward selection and backward elimination approach described in Bauer et al. (2009) was used to reduce the number of false-positive QTL. The false discovery rate (FDR) was set to *p* < 0.05 for the iterative multi-locus approach in the QTL model. In order to further reduce the number of false positive QTLs, we applied fivefold cross-validation procedure. he best linear unbiased estimators (BLUE) for the following traits and for each genotype were previously generated by Voss-Fels, et al.^37^: grain yield (GY), kernels per spike (KPS), kernels per m^2^ (KPM), harvest-index (HI), plant height (PH), heading date (HD), kernel crude protein (KCP), sedimentation value (SD), falling number (FN), protein yield (PY), Nitrogen-use efficiency (NUE), green canopy duration (GCD), radiation interception efficiency (RIE).

The figures were produced using the R package *“ggplot2”*^56^. The R package “*adegenet”*^49^ was used to produce the DAPC plot.

### Legal statement

The present study complies with relevant institutional, national, and international guidelines and legislation. Seeds of the bread wheat cultivars were provided by Leibniz Institute of Plant Genetics and Crop Plant Research (IPK) with permission to collect the seeds of the plant material by accepting the terms and conditions of the Standard Material Transfer Agreement (SMTA) of the International Treaty on Plant Genetic Resources for Food and Agriculture (http://www.fao.org/plant-treaty/).

## Results

### Genotyping analysis

The diversity panel comprising 221 genotypes was genotyped using the Infinium iSelect 15 K SNP chip and the 135 K Axiom Exome Capture Array. A total of 81,655 SNP marker were aligned to the wheat reference genome assembly RefSeq v1.0^25^, of which 54,389 markers aligned to physical positions on the assembled pseudomolecules. After stringent filtering for missing data and minor allele frequency we retained 25,510 SNP markers. However, aligning the markers to the reference genome without taking into account LD with the neighboring markers lead to putative mismapping of 11.7% of the markers. Following the LD-correction approach, the positions of 1388 SNP markers were corrected, whereas 1294 (5.1%) of markers showing ambiguous LD patterns or putative localization to multiple chromosomes were removed from the set. An example of marker misplacement is shown in Fig. 1. Performing GWAS for a simulated data set showed unexpected/spurious QTL on chromosome 1A with high logarithm of odds (LOD) scores. Unexpectedly, adjacent markers (physical distance 1395 bp) are in low LD with the main QTL and show considerably lower LOD scores (Fig. 1A and C). However, the LD-based correction approach suggests the correct location of this marker is on chromosome 1B, where it constitutes a haploblock of eight markers with high LD (Fig. 1D). Subsequent repetition of GWAS with the corrected map assigns the QTL to chromosome 1B surrounded by the markers of the haploblock (Fig. 1B).

**Figure 2:**
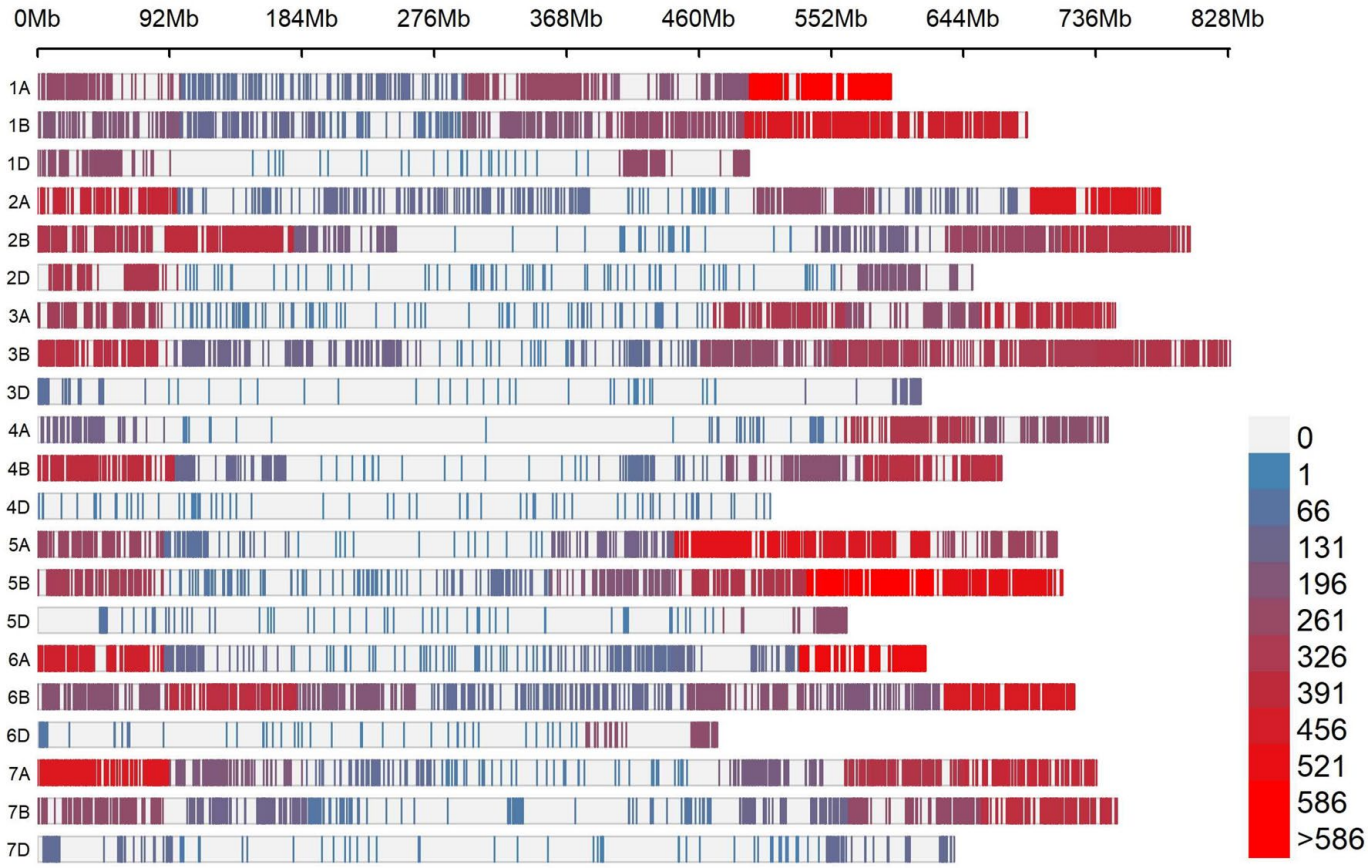
SNP-density plot of the 21 chromosomes of wheat; colors represent number of SNP within 100 Mbp.

Consequently, the LD-corrected map containing 24,216 SNP markers was used for further analysis (For the marker data and the LD-corrected map see Supplementary Table S2; for the genotypic data with imputations for missing marker data see Supplementary Table S3). Finally, 60% of markers (14,492) originated from the 135 K genotyping array, whereas 40% (9724 markers) originated from the 15 K genotyping array. The majority of the markers were localized on the A subgenome (9963 markers) and B subgenome (11,882 markers), whereas 10% of the markers were found on the D subgenome (2 371 markers). Furthermore, the A and B subgenomes had higher marker density per Mbp than the D subgenome, with 2.0 and 2.3 SNP/Mbp, respectively, compared to an average of 0.6 markers per Mbp (Fig. 2; Supplementary Table S4).

### Analysis of population structure

**Figure 3:**
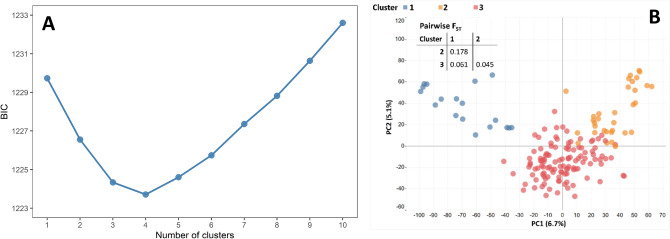
Estimation of optimal number of clusters and principal component analysis (PCA) of the German panel based on the marker data. (**A**) Cluster analysis with Bayesian Information Criterion (BIC) of K-means cluster on the x-axis and y-axis the number of estimated clusters, (**B**) PCA plot of the first two principal components. Colors indicate the three sub-populations identified with the elbow method.

Population structure was assessed using the full set of 24,216 SNP markers for cluster analysis using a K-means approach and principal component analysis (PCA). The cluster analysis assigned the accessions into three clusters following the elbow method (Fig. 3).

**Figure 4:**
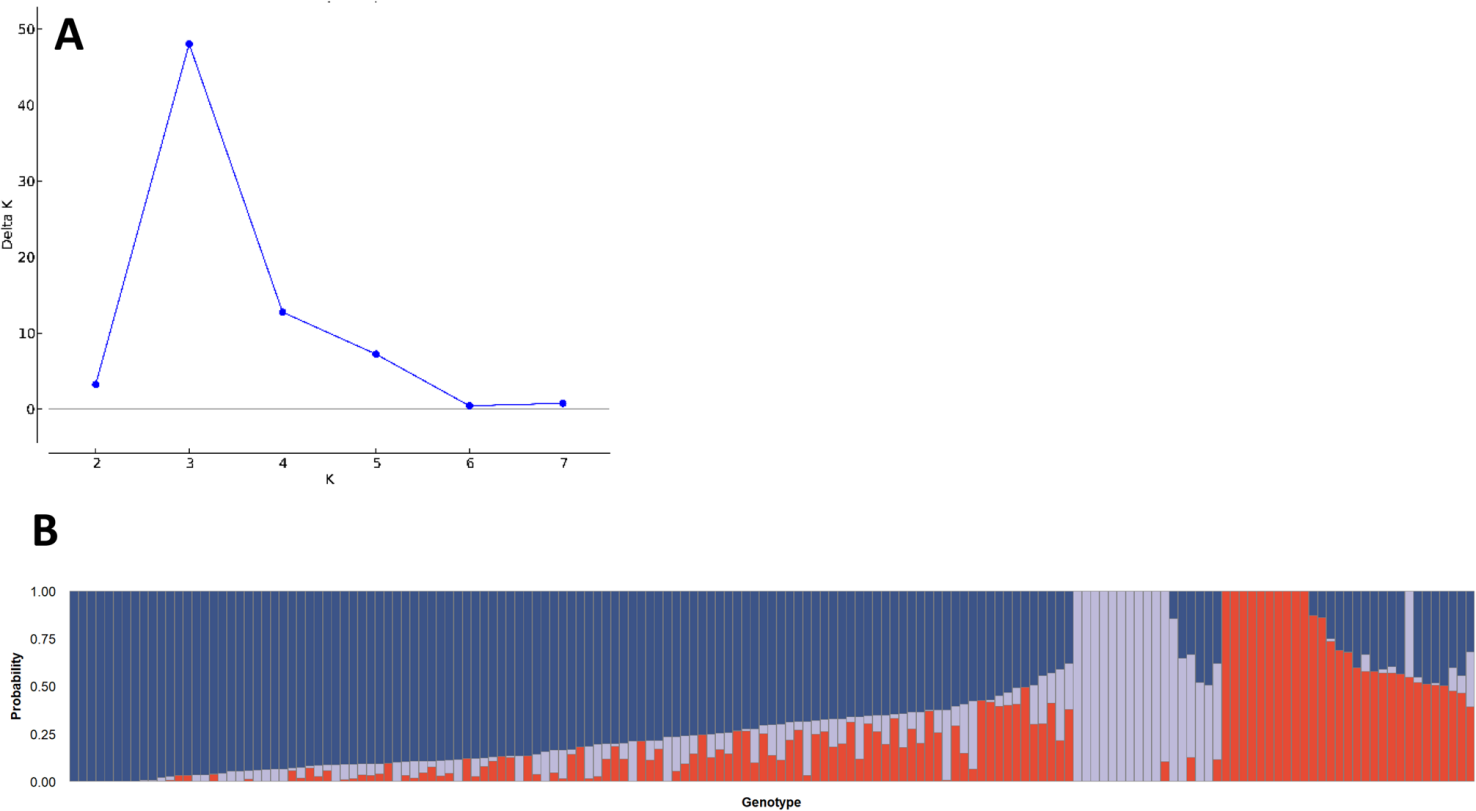
Population structure analysis of the German Panel using the software STRUCTURE. (**A**) Delta K calculated according to Evanno et al. (2005). (**B**) STRUCTURE bar plot showing the genetic clustering of the 165 German cultivars; each vertical bar represents an individual and the colors represent the different genetic components.

The first and second principal components of the PCA showed rather low explanatory power with respect to the genotypic variation of the analyzed accessions (6.7 and 5.1%, respectively). Stronger genetic divergence was observed between clusters 1 and 2 of the German panel (F_ST_ = 0.18). Population structure analysis using the software STRUCTURE suggested K = 3 for the German population (Fig. 4).

### Analysis of population structure in the context of 50 years of breeding history

Discriminant Analysis of Principal Components (DAPC) was conducted to investigate the relationship between the German winter wheat cultivars relative to their breeding history. Hence, the German cultivars were assigned into six groups according to the decade in which the variety was released (1960s, 1970s, 1980s, 1990s, 2000s and 2010–2020).

**Figure 5:**
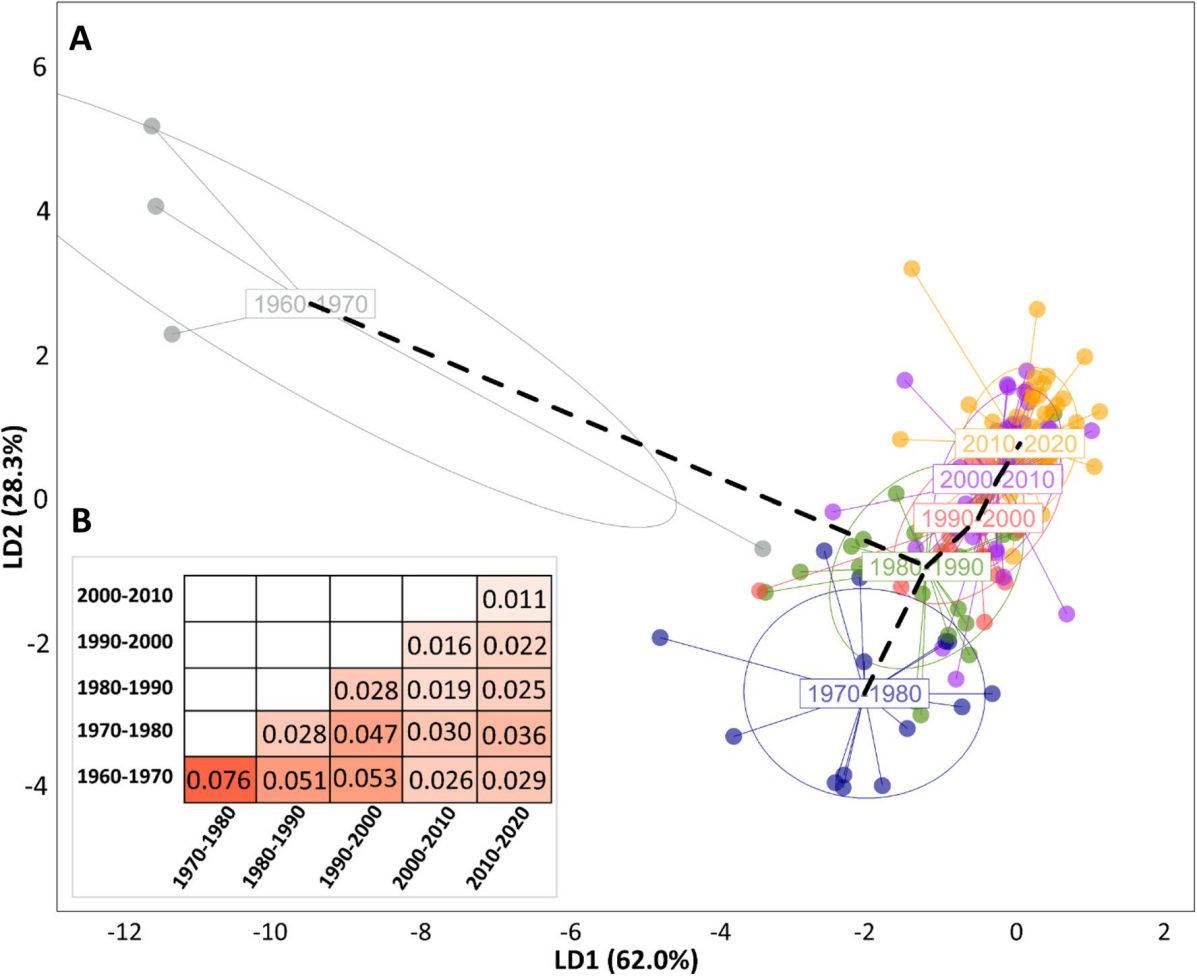
Discriminant Analysis of Principal Components (DAPC) and correlation matrix of FST differentiating between the German cultivars in the frame of their year of release. (**A**) DAPC scatterplot showing the individuals of the German population clustered in 6 groups according to the year of admission grouped into decades (from 1960–2020). Each dot represents single individuals of the population. LD1 and LD2 are the linear discriminant functions 1 and 2, respectively. (**B**) Heatmap of Nei's pairwise FST for the genotypes grouped into decades from 1960–2020.

Some grouping of German wheat cultivars by decade of release was apparent (Fig. 5). Specifically, the cultivars show some chronological separation with greater distances between genotypes released further apart in time. The first decade (1960–1970) was distinct from other decades, indicating a stronger differentiation between the genotypes released between 1960 and 1970 and newer genotypes. Notably, the distance between the clusters is shrinking over time, indicating higher genetic similarity among the cultivars registered during the recent decades.

Nei's pairwise F_ST_ also supported a greater differentiation between cultivars released in the 1960s and cultivars released in other decades, with the greatest similarity between cultivars released in modern decades Fig. 5.

**Figure 6:**
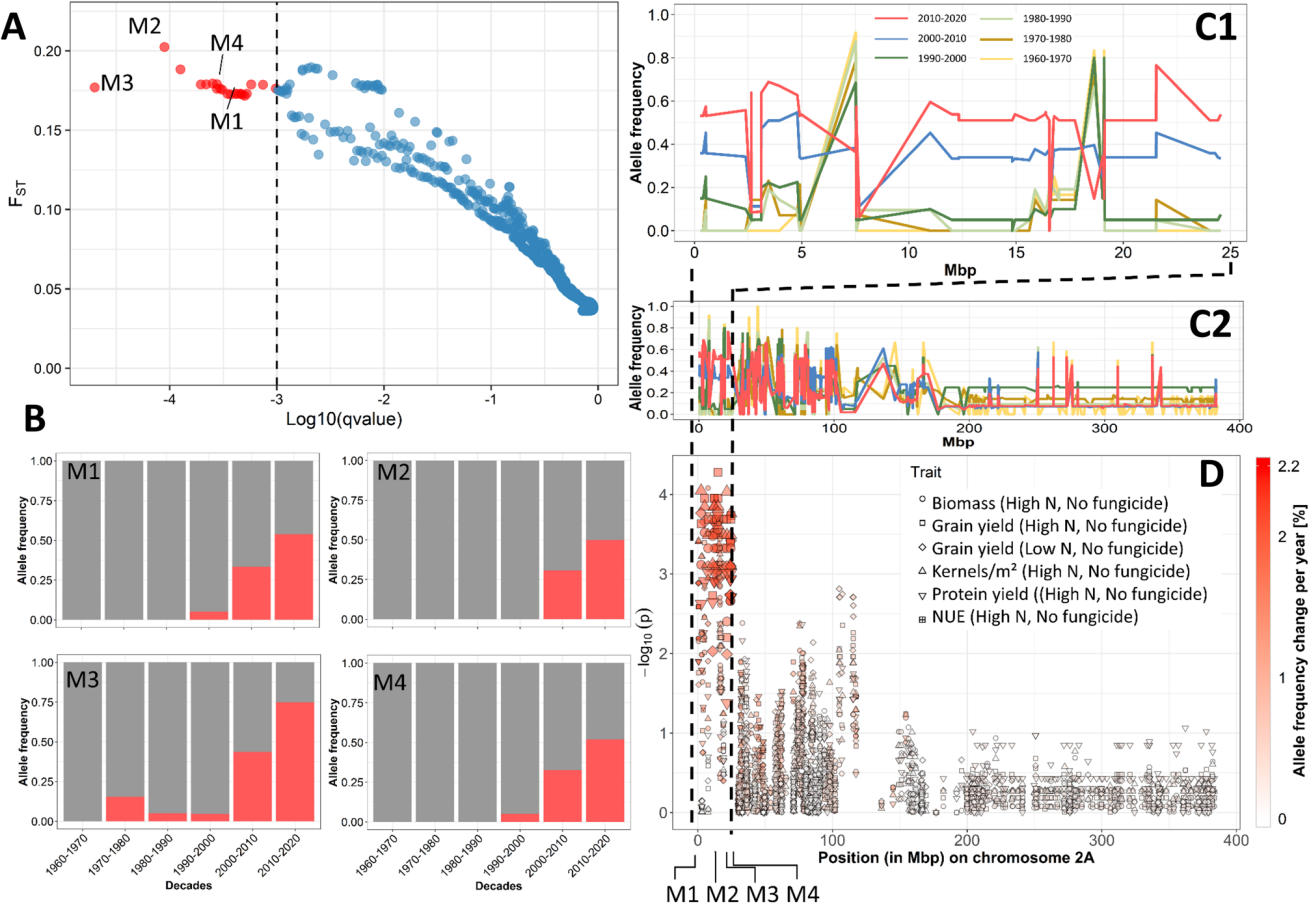
(**A**) Outlier loci identified by BayeScan for the German cultivars divided into six groups according to their decade of registration. FST is plotted against Log10(qvalue). The dashed vertical line indicates the significance threshold (FDR < 0.001). The significant outlier loci are highlighted in red. (**B**) Average allele frequency of selected markers (M1 to M4) among the six groups of wheat cultivars representing the decades of registration. (**C**) Average allele frequency of minor alleles on chr. 2A (C1 < 25 and C2 < 400 Mbp) of the cultivars grouped according to their decade of registration. (**D**) Manhattan plot showing marker trait associations on chromosome 2A (< 400 Mbp) for biomass, major agronomic and physiological traits (for further information see Voss-Fels et al.^37^. The x-axis is showing the chromosomal position in bp, the y-axis is showing the  − log(*p*-value). The color intensity is showing the annual allele frequency change [%] of the specific SNP markers.

Subsequently, an outlier locus detection approach was applied to detect signatures of breeding among the German winter wheat cultivars registered between 1960 and 2020. Accordingly, the software *BayeScan* v2.1 was utilized to scan for genomic regions which were putatively affected by breeding and therefore experiencing changes of allele frequency in the German population. Setting the FDR < 0.001 BayeScan identified 22 outlier loci (Fig. 6A). A total of 18 outlier loci were detected on chromosome 2AS spanning a 22.2 Mbp region between the SNP marker AX-158573264 (2,380,829 bp, denoted as M1) and SNP marker AX-158522761 (24,543,742 bp, denoted as M4). The second region harboring three outlier loci was detected on a comparably small genomic region of 0.2 Mbp on chromosome 2Bs between the SNP marker Ra_c2110_494 (146,735,843 bp) and the SNP RAC875_c14105_66 (146,902,161 bp). Surprisingly, the minor allele frequency for the markers between M1 and M4 was strongly shifted from 0% in the old genotypes (registered 1960–1970) to 50–75% in modern varieties registered between 2010 and 2020 (Fig. 6B). The same pattern of allele frequency shift for the markers between M1 and M4 is shown in Fig. 6C. Subsequently, GWAS was performed using precalculated BLUE values of several agronomic and physiological traits obtained from multi-year field trials under two nitrogen levels and with and without fungicide treatment^37^. The GWAS results indicate a QTL hotspot between M1 and M4 on chromosome 2AS (Fig. 6D). The annual allele frequency change in this hotspot is above 2% and much higher than that of other regions on chromosome 2AS. In contrast, no significant marker by trait interaction was detected for the outlier loci detected on chromosome 2BS, indicating that this region has a minor effect on the analyzed traits.

The summary of GWAS results is presented in (Supplementary Table S5). Notably, the highest significant outlier loci M3 (Excalibur_c21663_145) and M2 (Tdurum_contig63196_123) were linked to a NAC domain and to ATP-sulfurylase PUA-like domain, respectively. Both domains are linked to plant responsive genes towards biotic stress factors, including pathogens.

### Further investigation of the M1–M4 selective sweep region

**Figure 7:**
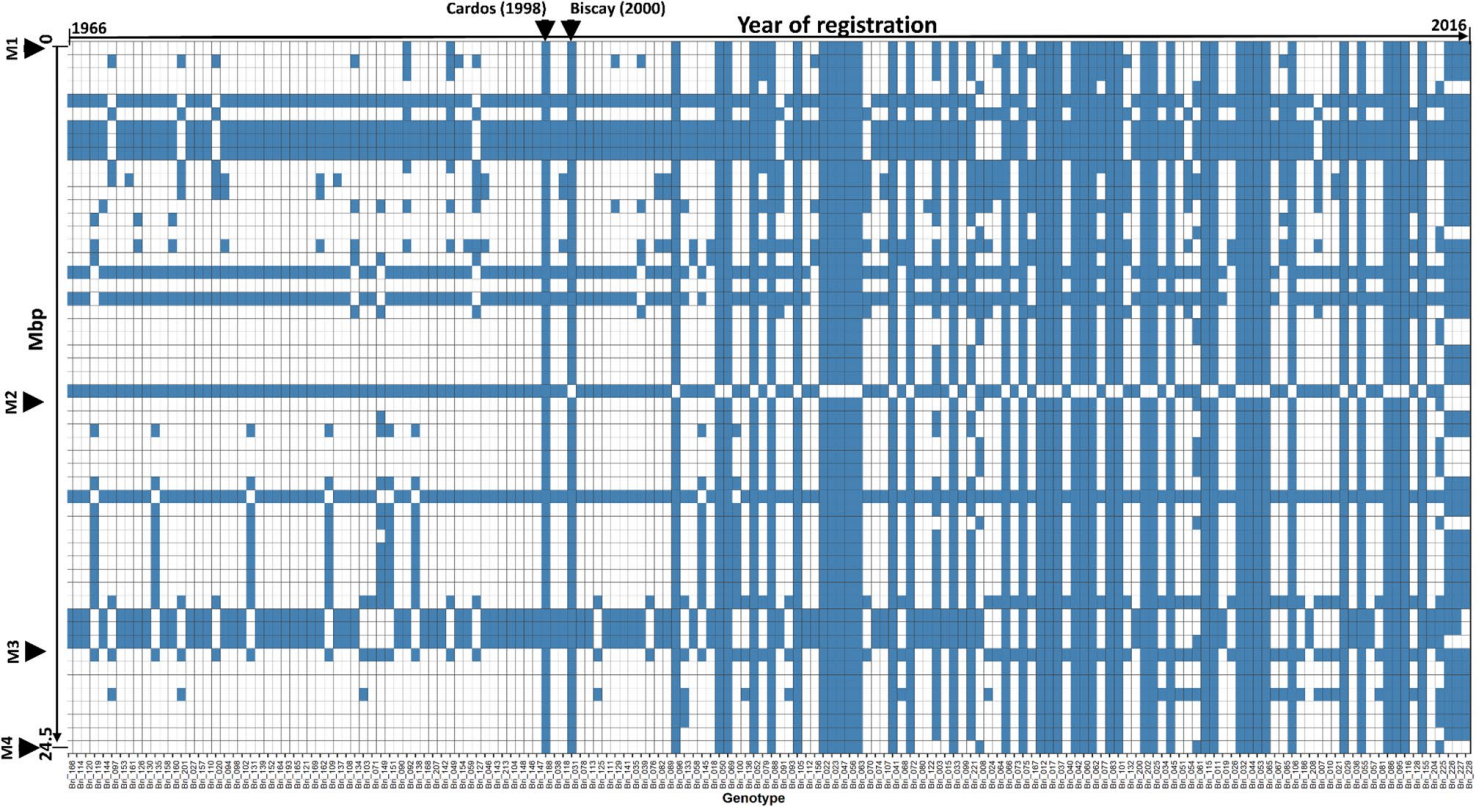
Graphical genotypes of chromosome 2AS (0–24.5 Mbp) phased according to the cultivar ‘Cardos’ containing the 2NS/2AS translocation across the genotypes of the German panel. Each horizontal bar represents the alleles for each of the 54 SNPs on the region 0–24.5 Mbp (y-axis). Each vertical bar represents genotypes of the German panel ordered according to their year of registration from left to right (x-axis).

The identified selective sweep between M1– M4 is known to be the target of an introgression from wheat wild relative species *Aegilops ventricosa*, known as the 2NS/2AS translocation^57^. Among the German panel the cultivar 'Cardos' was the first genotype containing the 2NS/2AS translocation. Since the release of 'Cardos' in year 1998 the numbers of the cultivars containing this introgression increased successively (Fig. 7).

**Figure 8:**
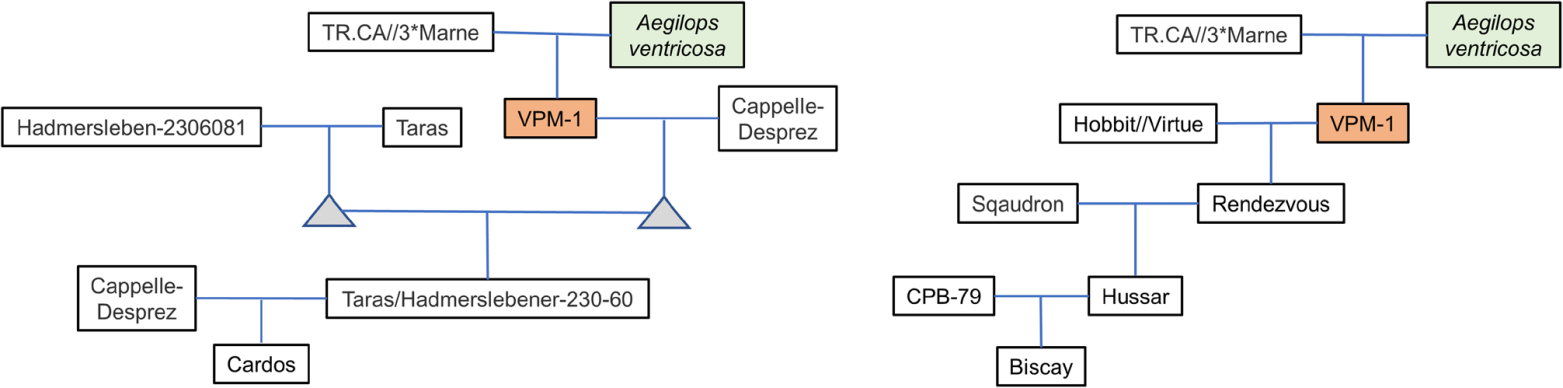
Pedigree tree of the German wheat cultivars ‘Cardos’ and ‘Biscay’ containing 2NS/2AS translocation descending from Aegilops ventricosa via VPM-1 ( Source: http://wheatpedigree.net/).

The cultivar 'Biscay' registered in year 2000 was the second genotype containing this introgression. Studying the pedigree information for the cultivars ‘Cardos’ and ‘Biscay’, it is obvious that the genotype VPM-1 containing the 2NS/2AS translocation is the common ancestor of both genotypes (Fig. 8).

Ultimately, the BLUE values of traits that were showing significant QTL under the treatment “without fungicide application” (see Fig. 6D) were visualized in combination with the presence/absence information of the 2NS/2AS translocation (Supplementary Figure S1). Due to the polygenic nature of the analyzed traits the presence of the 2NS/2AS translocation is not essential. However, in most cases recently registered genotypes containing the 2NS/2AS translocation show higher performance than older genotypes without the translocation.

## Discussion

### LD-based correction of marker positions

Advances in sequencing and genotyping technologies have significantly increased the number of available molecular markers in many crop species. The availability of high-quality reference genomes for major crops like maize, rice, potato, cassava and wheat are great leaps forward in genome research facilitating plant breeding^58^. In this study we aligned the 25,510 SNP markers obtained from genotyping a diversity panel consisting of 221 winter wheat cultivars to the reference wheat genome assembly RefSeq v1.0^25^. However, we observed a high number of markers with anomalously high LD with markers on other chromosomes but low LD with neighboring markers on the same chromosome. Unusual LD behavior of SNP markers is a serious impediment for studies, such as GWAS, that are dependent on accurate positions of applied markers for candidate gene identification^44,59^. Following the LD correction approach, we were able to assign 5.4% of the misplaced markers to corrected physical positions. With the set of 24,216 remaining markers with corrected positions we ran population analysis for the diversity panel containing 221 winter wheat genotypes as well as the panel of 165 German cultivars.

Even though there are other confounding factors affecting LD anomalies (e.g. population stratification), one major source of inconsistency in LD patterns is due to problems with the reference genome assembly. Similar observations were previously described by Money et al.^59^, who also observed a false positive QTL for skin color intensity in apple (*Malus domestica* Borkh.) on chromosome 3, where a marker was in low LD with nearby SNPs on chromosome 3 but in high LD with markers on chromosome 9. The authors concluded that 10–20% of SNPs in their apple data set had incorrect physical coordinates due to the available reference assembly. In hexaploid wheat, the high content of repetitive elements as well as high similarity among the different genome components are major obstacles for the construction of the reference genome assembly^60,61^. Moreover, the importance of structural variants for construction of genome assemblies is becoming increasingly clear^62,63^. Accordingly, studying structural variation in the human reference genome, Audano, et al.^64^ concluded that insufficiencies and errors in the human reference genome demand for additional reference genomes to include SV by construction of the human pan-genome. Although the fully annotated reference genome of bread wheat variety 'Chinese Spring' is the current gold standard, the large and complex genome of hexaploid^65^ wheat, consisting of three homoeologous and highly repetitive subgenomes, makes it difficult to construct a high-quality reference genome^66^. Additionally, Walkowiak et al.^65^ pointed out on the large genetic distance of the variety 'Chinese Spring' with the Western breeding material. In future, correction of reference genome assemblies based on the pangenome as well as using different types of mapping populations may prove useful in improving this important genomic resource^67^.

### Analysis of population structure in the frame of breeding history

Population structure analysis allowed us to detect breeding signatures in the German panel of wheat cultivars representing 50 years of breeding history. Discriminant Analysis of Principal Components (DAPC) suggested reduced genetic variability in German winter wheat cultivars registered between the year 2000–2010 and the cultivars registered after 2010. This is in line with previous reports, underlining that the intensive selection in modern plant breeding programs within a narrow range of plant germplasm with limited allele introgressions over time is the main cause of loss of genetic diversity^68,69^. This is not surprising, as in many cases breeders focus on only a few best-performing varieties, neglecting genetic diversity^70^. Similarly, Voss-Fels, et al.^37^, examining the same group of genotypes, concluded that breeding has gradually reduced the number of negative or neutral haplotypes in recent decades by focusing on genotypes with favorable haplotypes. Moreover, modern cultivars outperform older cultivars with respect to morphological, physiological as well as agronomic traits under low-input conditions e.g. without fungicide treatment^37,38^.

### Detection of breeding signatures in German winter wheat

The F_ST_-based Bayesian genome scan approach implemented in 'BayeScan' is frequently used for detection of candidate loci under selection by estimating the locus effect as well as the population effect^71–73^. Using the software 'BayeScan' we were able to detect signals of directional selection in the German cultivars in the course of 50 years of breeding. The identified segment on chromosome 2AS, which was undergoing major effective changes, shows pattern of selective sweeps among the German cultivars registered during last decades. The SNP markers between M1 and M4 of the identified region on chromosome 2AS showed remarkable shift of allele frequencies of the minor allele from 0% to more than 50% among the genotypes of the German panel registered between 1960 and 2020. Similar observations of annual allele frequency changes of outlier loci were reported by Fu and Somers^74^ and N’Diaye, et al.^73^ studying the breeding history of Canadian spring wheat and durum wheat, respectively. Remarkably, the identified region between M1 and M4 is known to be subject to a large introgression from chromosome 2NS of *Aegilops ventricosa* Tausch to chromosome 2AS of the wheat line VPM-1^18^. The 2NS/2AS translocation is known to harbor large number of genes with resistance against yellow rust, brown rust, powdery mildew, eyespot disease and wheat blast disease^18–20,57,75–77^. Several reports underline the superiority of genotypes containing this translocation with respect to resistance towards multiple pathogens, among them the cultivar 'Cardos' which was the first cultivar among the German panel containing the 2NS/AS translocation^19,57,78,79^. The cultivar ‘Cardos’ was also for many years the reference resistance genotype among German wheat varieties^80^. Recently^65^, applied genotyping by sequencing approach to detect the 2NS/2AS translocation in three wheat panels. In agreement with our observations, they concluded that the presence of this introgressions was associated with disease resistance and higher grain yield.

Due to the directional selection the identified selective sweep on chromosome 2AS was not only subject to major shift of allele frequency. Additionally, GWAS analysis revealed significant marker by treatment interactions of this region linked to plant responsive genes related to biotic stress factors. Positive or directional selection and selective sweeps in natural populations as well as in long-term breeding approaches (historical breeding programs) have been the focus of population geneticists for many years^74,81,82^. Linking shift of allele frequency with QTL results was previously reported for crops like barley^83^ and maize^84^. However, to our knowledge the present study is among few attempts to link results from GWAS analysis with the directional change of allele frequency in a historical association panel of wheat^85^. Finally, as high-throughput genotyping now allows us to genetically characterize large numbers of genotypes with low costs, now the focus has shifted towards reduction of phenotyping costs. In this frame, identification of beneficial selective sweeps by studying changes of allelic frequency in a historical association panel and their integration into breeding programs will help to reduce phenotyping costs and increase breeding efficiency^86,87^. The detected 2NS/2AS translocation, containing effective genes related to disease resistance, should be considered as valuable source of resistance genes for allele mining approaches to produce future varieties with higher disease resistance.

## Conclusions

The LD-corrected physical genome sequence of wheat helps to enhance the power of genome wide association studies as well as identification of candidate genes in wheat. Additionally, we were able to trace back the translocation of 2NS/2AS of *Aegilops ventricosa* Tausch to the cultivar ‘Cardos’ (registered in 1998), which was the first German cultivar among the German panel containing this translocation. Moreover, understanding breeding from the historical perspective by screening for selective sweeps (among the genotypes of a historical association panel) offers an alternative for identifying favorable QTL-regions by means of population genetics, even without phenotyping.

## Supplementary Information

Supplementary Figure.

Supplementary Table 1.

Supplementary Table 2.

Supplementary Table 3.

Supplementary Table 4.

Supplementary Table 5.

## Data Availability

The datasets supporting the conclusions of this article are included within the article and its additional files.
