# Supplementary material for: Detection of breeding signatures in wheat using a linkage disequilibrium-corrected mapping approach
Source: Sci Rep. 2021 Mar 9;11:5527. doi: 10.1038/s41598-021-85226-1 (PMC7970893; doi:10.1038/s41598-021-85226-1)
Supplement: Supplementary file 1 — Supplementary Figure. [file 41598_2021_85226_MOESM1_ESM.docx]

Supplementary Information for

**Detection of breeding signatures in wheat using a linkage disequilibrium-corrected mapping approach**

Said Dadshani^1^, Boby Mathew^2^, Agim Ballvora^1^, Annaliese S. Mason^1^, Jens Léon^1^

Institute of Crop Science and Resource

Conservation (INRES), Plant Breeding,

University of Bonn, Bonn, German

^1^ Institute of Crop Science and Resource Conservation (INRES), Plant Breeding, University of Bonn, Bonn, Germany

^2^ Bayer CropScience, Monheim am Rhein, Germany

Corresponding authors:
Dr. Said Dadshani

E-mail: [dadshani@uni-bonn.de](mailto:dadshani@uni-bonn.de)

Tel: +49-228-73-3259

Prof. Dr. Jens Léon

E-mail: j.leon@uni-bonn.de

Tel: +49-228-73-3259


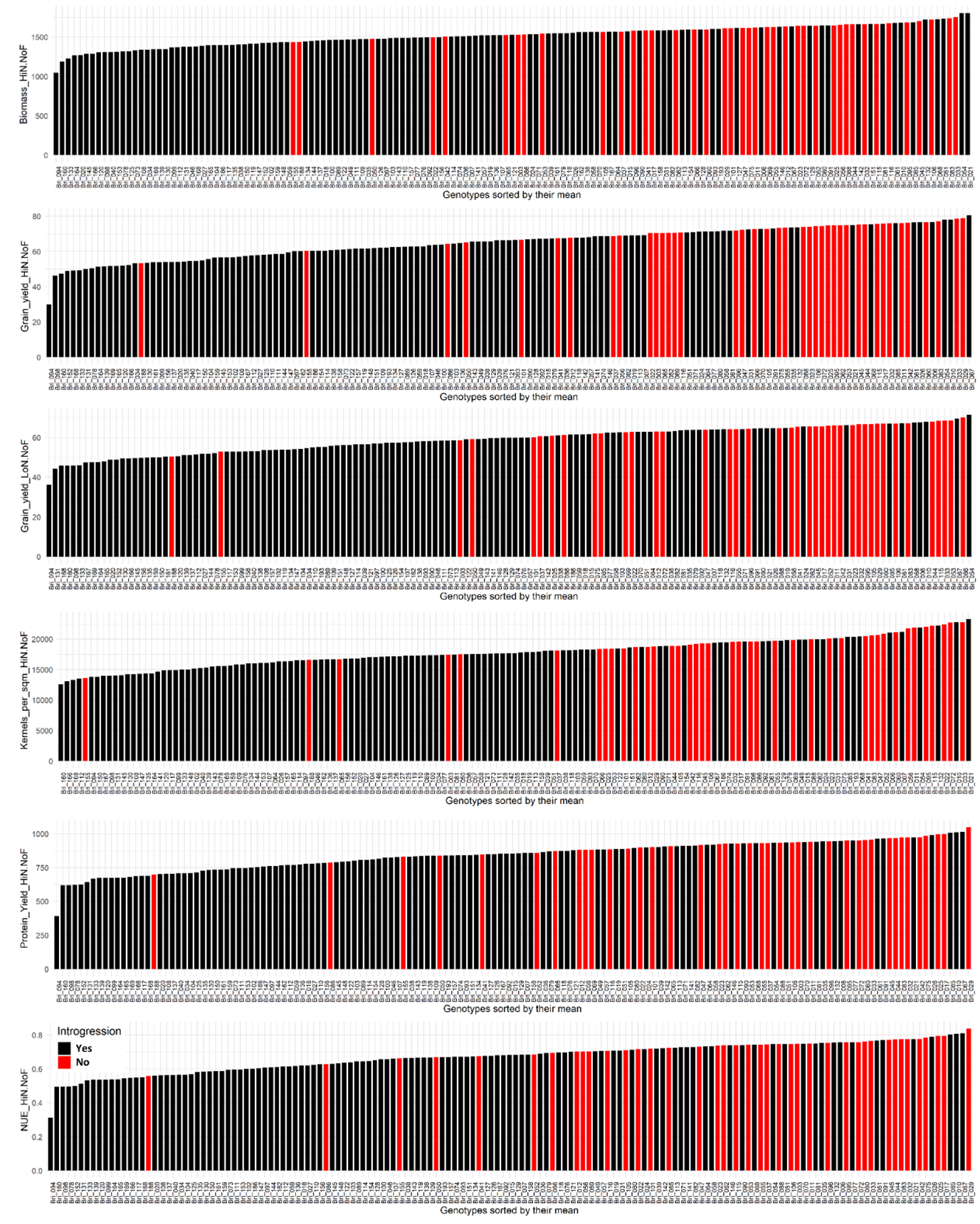


**Figure S1:** Bar plot of best linear unbiased estimator (BLUE) of field data without application of fungicides. A) biomass, B) grain yield, grain yield, C) kernel/m², D) protein yield, E) Nitrogen-use efficiency. For further information see Voss-Fels et al. (2019). Genotypes are sorted according to the highest BLUE value. Genotypes containing the 2NS/2AS translocation are highlighted in red; genotypes not containing the translocation are highlighted in black.
